# FBXW8-dependent degradation of MRFAP1 in anaphase controls mitotic cell death

**DOI:** 10.18632/oncotarget.21843

**Published:** 2017-10-12

**Authors:** Duan-Zhuo Li, Shun-Fang Liu, Lan Zhu, Yu-Xing Wang, Yi-Xiang Chen, Jie Liu, Gang Hu, Xin Yu, Jian Li, Jin Zhang, Zhi-Xiang Wu, Han Lu, Wei Liu, Bin Liu

**Affiliations:** ^1^ Hubei Key Laboratory for Kidney Disease Pathogenesis and Intervention, Huangshi Central Hospital of Edong Healthcare Group, Hubei Polytechnic University School of Medicine, Huangshi, Hubei 435003, PR China; ^2^ Department of Oncology, Tongji Hospital of Tongji Medical College, Huazhong University of Science and Technology, Wuhan 430030, PR China; ^3^ School of Molecular Sciences and Biodesign Center for Applied Structural Discovery, Biodesign Institute, Arizona State University, Tempe, AZ 85287, USA; ^4^ Department of Molecular and Cellular Biochemistry, University of Kentucky, Lexington, KY 40536, USA; ^5^ School of Basic Medical Sciences, Nanchang University, Nanchang, Jiangxi 330031, PR China; ^6^ Department of Pediatric Surgery, Xinhua Hospital, Shanghai Jiao-Tong University School of Medicine (SJTU-SM), Shanghai 200092, PR China; ^7^ Department of Anesthesiology, Ruijin Hospital, Shanghai Jiao-Tong University School of Medicine (SJTU-SM), Shanghai 200025, PR China

**Keywords:** FBXW8, genomic instability, MRFAP1, mitosis

## Abstract

Mof4 family associated protein 1 (MRFAP1) is a 14 kDa nuclear protein, which involves in maintaining normal histone modification levels by negatively regulating recruitment of the NuA4 (nucleosome acetyltransferase of H4) histone acetyltransferase complex to chromatin. MRFAP1 has been identified as one of the most up-regulated proteins after NEDD8 (neural precursor cell expressed developmentally down-regulated 8) inhibition in multiple human cell lines. However, the biological function of MRFAP1 and the E3 ligase that targets MRFAP1 for destruction remain mysterious. Here we show, by using an immunoprecipitation-based proteomics screen, that MRFAP1 is an interactor of the F-box protein FBXW8. MRFAP1 is degraded by means of the ubiquitin ligase Cul7/FBXW8 during mitotic anaphase-telophase transition and accumulated in mitotic metaphase. Overexpression of FBXW8 increased the polyubiquitination and decreased the stability of MRFAP1, whereas knockdown of FBXW8 prolonged the half-life of MRFAP1. Moreover, forced expression of MRFAP1 in HeLa cells caused growth retardation and genomic instability, leading to severe mitotic cell death. Thus, Cul7/FBXW8-mediated destruction of MRFAP1 is a regulatory component monitoring the anaphase-telophase transition and preventing genomic instability.

## INTRODUCTION

The specific, rapid, and temporally controlled proteolysis of cellular regulators by the ubiquitin-proteasome system (UPS) determined the unidirectional progression through the cell cycle [1]. The specificity of this system is mainly determined by E3 ubiquitin ligases [2]. The Cullin-Ring Ligases (CRL) are the well-studied RING E3 ubiquitin ligases, among which the Skp1-Cullin1-Fbox (SCF) complex has been extensively characterized. To date, the Cullin family contains eight mammalian proteins, Cul1, Cul2, Cul3, Cul4A, Cul4B, Cul5, Cul7 and Cul9 [3].

The SCF complexes compose of the scaffold protein Cul1, the RING finger protein Rbx1 and the adaptor protein Skp1 to bridge variable F-box proteins that determine substrate recognition and recruitment to the SCF complexes [4]. The human genome encodes more than 70 F-box proteins that contain a homologous 40-amino-acid F-box domain which can be further divided into three major classes: the FBXW family, the FBXL family, and the FBXO family [5]. Among these F-box proteins, FBXW8 is not only the receptor component of an SCF complex, but also the unique F-box protein known to interact with Cul7 [6]. Cul7 interacts directly with FBXW8 and indirectly with Skp1 in an FBXW8-dependent manner [6]. Interestingly, Cul7 has higher affinity with FBXW8 than Cul1, and the placental phenotypes of FBXW8^-/-^ and Cul7^-/-^ mice are similar, indicating that Cul7 forms a ubiquitin E3 ligase complex with FBXW8 to target substrates for destruction [7]. In agreement with that, Cul7/FBXW8 ubiquitin ligase-mediated HPK1 (hematopoietic progenitor kinase-1) degradation played a critical role in cell proliferation and differentiation [8].

Mof4 family associated protein 1 (MRFAP1) is highly conserved and only presents in mammals, which is consistent with its specific role in the regulation of mortality factor 4 like 1 (MORF4L1) protein complexes. MRFAP1 maintains the normal histone modification levels by negatively regulating recruitment of the NuA4 histone acetyltransferase complex to chromatin [9]. It also regulates the transcription factors activities of the MRG family (Mas-related genes) via competing with MRGBP for binding to MORF4L1, and plays a role in spermatogenesis, possibly by regulating the hyper-acetylation of chromatin on histone H4 [10]. Recently, by quantitative proteomic analysis, MRFAP1 has been shown to be degraded by CRL via the ubiquitin-proteasome system [10, 11]. However, which CRL family member involved in this process remains unknown.

In this study, by using an immunoprecipitation-based proteomics screen, we identified that MRFAP1 was an interactor of FBXW8. MRFAP1 was a novel cell cycle-regulated protein degraded by Cul7/FBXW8 ubiquitin ligase during mitotic anaphase-telophase transition and accumulated in mitotic metaphase. Failure to degrade MRFAP1 in HeLa cells caused growth retardation and genomic instability, leading to severe mitotic cell death.

## RESULTS

### Proteomics screen identified MRFAP1 as a potential binding partner of Cul7/FBXW8 E3 ligase complex

**Figure 1:**
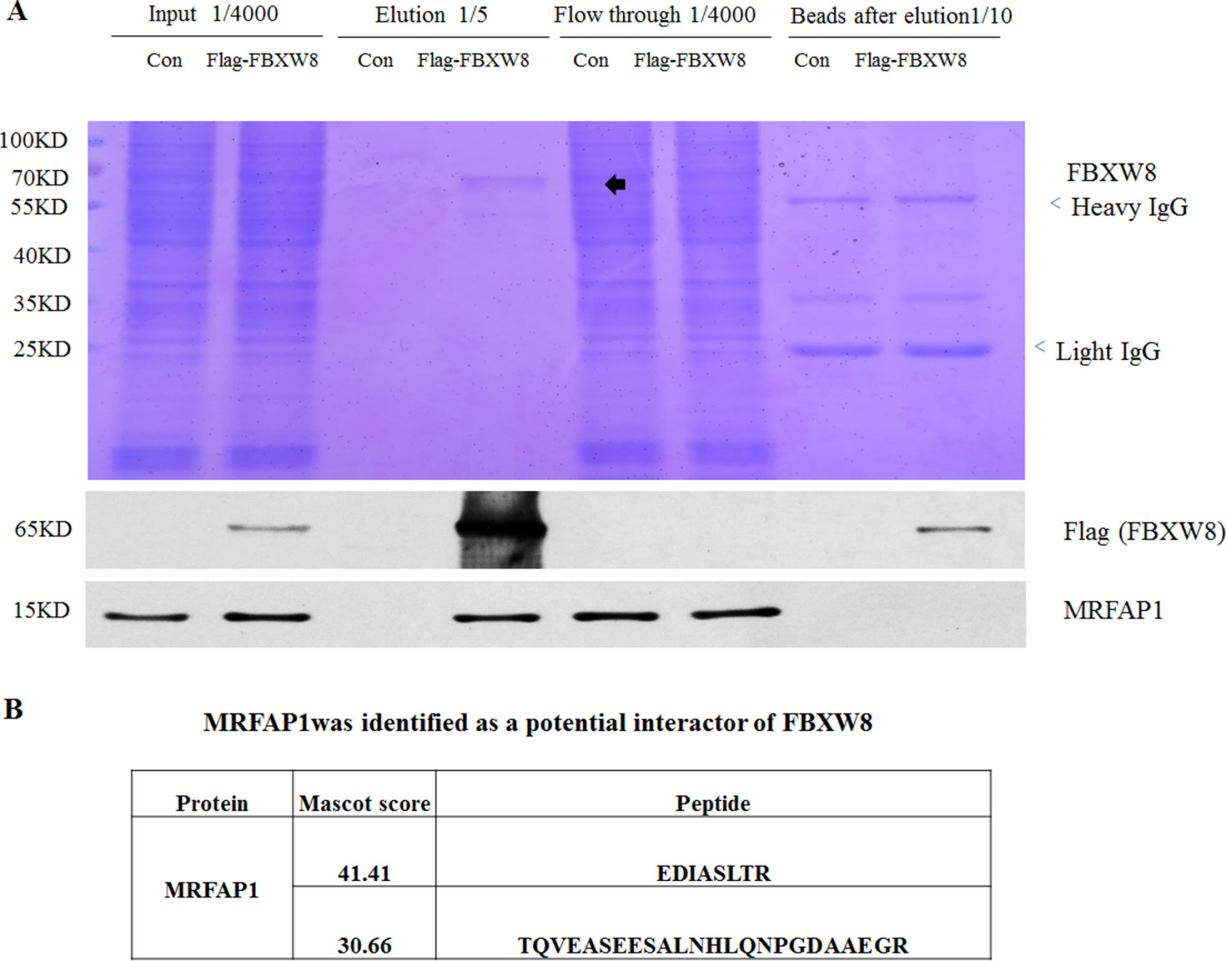
Proteomics screen identified MRFAP1 as a potential binding partner of Cul7/FBXW8 E3 ligase complex (**A**) After 4 hours 1 µM MLN4924 treatment, lysates from 293T cells stably expressing either Flag or Flag-FBXW8 were immunoprecipitated with anti-FLAG M2 resin. Bound proteins were eluted with FLAG peptide, and all separated cell lysate components were resolved on 10% SDS-PAGE gel, stained by Coomassie Brilliant or subjected to immunoblotting with the indicated antibodies. The gel was continuously cut and subjected to LC−MS/MS analysis. (**B**) MRFAP1 was identified as a potential binding partner of FBXW8. Two unique peptides were assigned to MRFAP1 from MLN4924 treated Flag-FBXW8 but not FLAG-control 293T cell line.

To systematically identify the candidate substrates of Cul7/FBXW8, we performed an immunoprecipitation-based proteomics screen in 293T cell stably expressing Flag-FBXW8. MLN4924, a potent and selective inhibitor of NEDD8-activating enzyme, which specifically disrupts CRLs-mediated protein turnover, was used to prevent the degradation of potential FBXW8 substrates and might enhance the protein-protein interactions with FBXW8. The cell lysate from Flag-control or Flag-FBXW8 293T cells with 4 hours MLN4924 treatment were subjected to immunoprecipitation with anti-Flag M2 resin to purify SCF-FBXW8 complexes. The whole IP procedure was monitored by running all separated cell lysate components on SDS-PAGE, followed by Coomassie Brilliant Blue staining (Figure 1A). The gel was continuously cut and subjected to LC−MS/MS analysis. The results indicate that Cul7 but not Cul1 was the highest score interacting protein of FBXW8 group (data not shown) as expected. Among a number of peptides from putative novel substrates, we recovered two unique peptides from MRFAP1 (EDIASLTR and TQVEASEESALNHLQNPGDAAEGR) (Figure 1B). The affinity efficiency for Flag-FBXW8 was very high, as almost all exogenously expressed FBXW8 was accumulated in the elution group (Figure 1A). Moreover, MRFAP1 was detected in the final elution of Flag-FBXW8 group and flow through of both groups (Figure 1A), suggesting that at least one part of endogenous MRFAP1 was specifically associated with FBXW8.

### MRFAP1 is co-localized and associated with FBXW8

**Figure 2:**
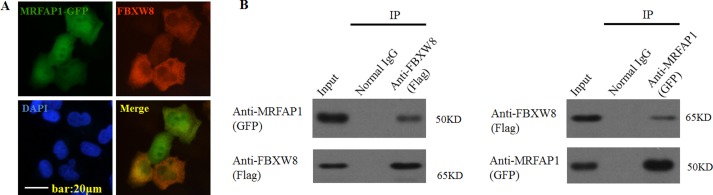
MRFAP1 is associated with FBXW8 (**A**) HeLa cells were co-transfected with GFP-MRFAP1 (green) and Flag-FBXW8 (red) vectors for 36 hours, cells were then fixed and stained with anti-Flag antibody. Cell nuclei were stained with DAPI. Immunofluorescence was performed with anti-flag antibody followed by Alexa Fluor 594-conjugated anti-mouse IgG antibody (red). Bar indicated 20 μm. (**B**) 293T cells were co-transfected with GFP-MRFAP1 and Flag-FBXW8 vectors for 36 hours, the cell lysates were subjected to immunoprecipitation with either GFP or Flag antibodies. Western blot was used to detect the interactions between FBXW8 and MRFAP1.

To determine the subcellular distribution of FBXW8, and further verify the spatio-temporal possibility that FBXW8 and MRFAP1 might interact with each other in mammalian cells *in vivo*, we utilized immunofluorescence microscopy on HeLa cells co-expression of Flag-FBXW8 and GFP-MRFAP1. As shown in Figure 2A, FBXW8 was distributed throughout the cytoplasm and less expressed in the nucleus of interphase cells. However, most MRFAP1 was distributed in the nucleus. Nevertheless, in agreement with our biochemistry data, the overlapping between FBXW8 and MRFAP1 in the nucleus was clearly observed (Figure 2A). Moreover, the interaction between exogenous FBXW8 and MRFAP1 was also confirmed by immunoprecipitation with either GFP or Flag antibodies (Figure 2B). Taken together, these data suggested that MRFAP1 was associated with FBXW8.

### FBXW8 regulates MRFAP1 ubiquitination and degradation

**Figure 3:**
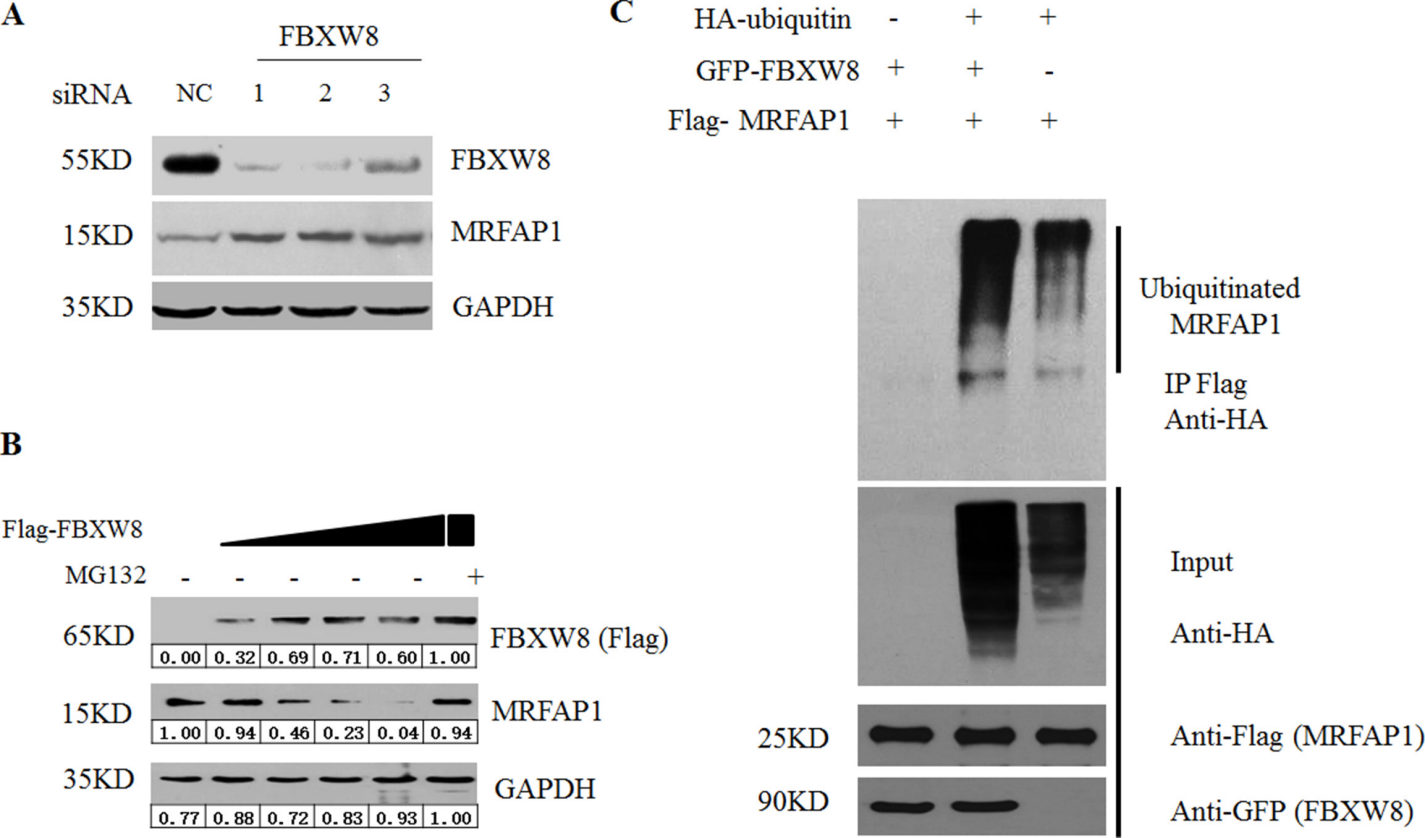
FBXW8 regulates MRFAP1 ubiquitination and degradation (**A**) 293T cells were transfected with negative control or three siRNAs against FBXW8 for 36 hours. The whole cell lysate was subjected to Western blot by the indicated antibodies. (**B**) 293T cells were transfected with increase dose of Flag-FBXW8 plasmids for 36 hours. 10 μM MG132 was added for 6 hours as indicated, and the whole cell lysate was subjected to Western blot by the indicated antibodies. (**C**) 293T cells were transfected with Flag-MRFAP1, GFP-FBXW8 or HA-Ubiquitin for 30 hours. After 6 hours MG132 treatment, samples were immunoprecipitated with anti-flag resin, and analyzed by immunoblotting with the indicated antibodies.

**Figure 4:**
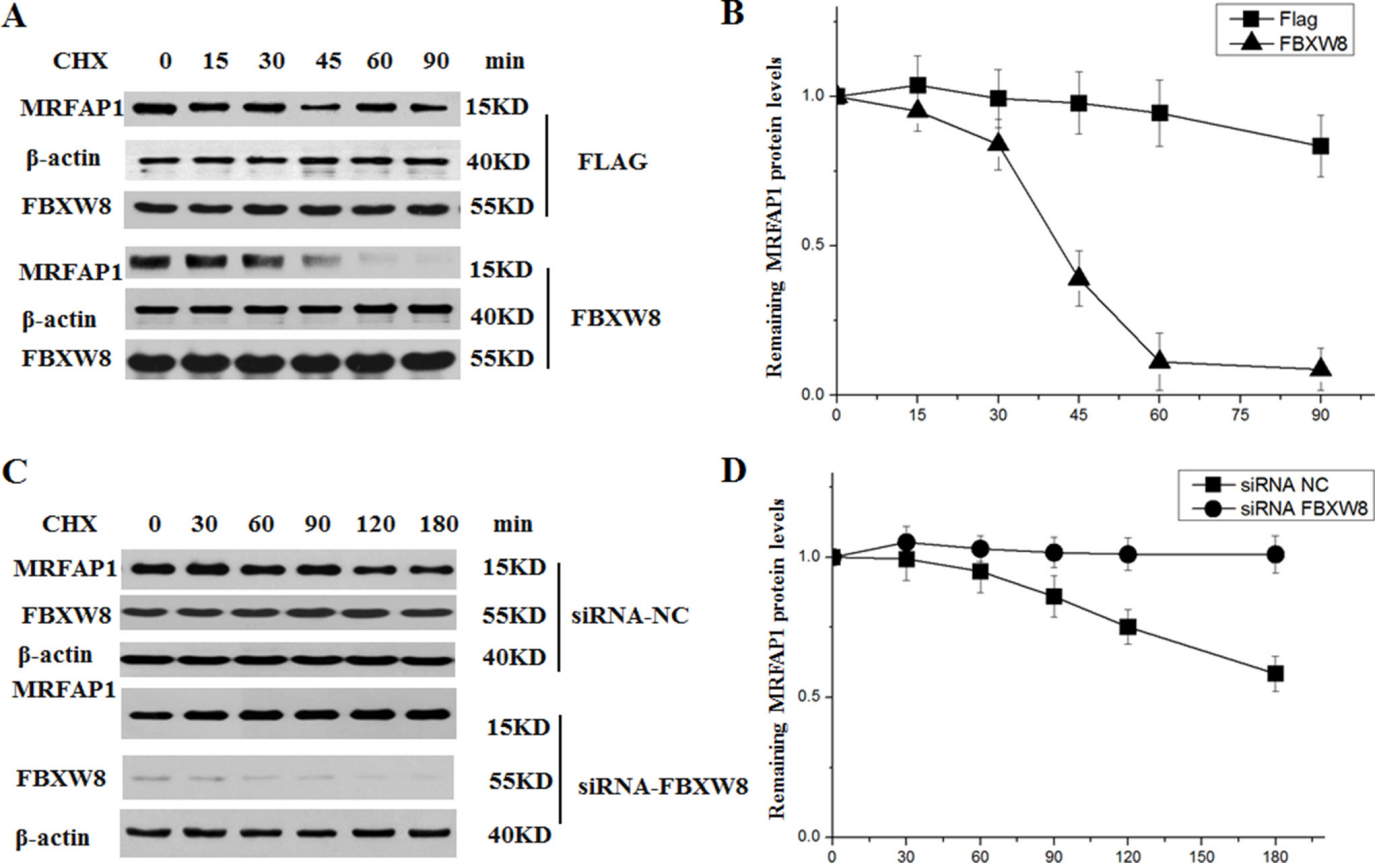
FBXW8 regulates the stability of MRFAP1 (**A**) 293T cells were transfected with empty vector or FBXW8 plasmids for 36 hours, 10 μM CHX was added for the indicated time. The whole cell lysate were detected by Western blot (A) using the indicated antibodies. (**B**) The intensity of the bands from the experiment shown on A was measured, and the ratio between the relative levels of MRFAP1 and β-actin in 0 hour was set as 1.0. (**C**) 293T cells were transfected with siRNAs targeting FBXW8 or negative control for 36 hours, 10 μM CHX was added for the indicated time. The whole cell lysate were detected by Western blot using the indicated antibodies. (*n* = 3 for each group). (**D**) The intensity of the bands from the experiment shown on C was measured, and the ratio between the relative levels of MRFAP1 and β-actin in 0 hour was set as 1.0.

Given the specific interaction between FBXW8 and MRFAP1, we then asked whether FBXW8 regulates the stability of MRFAP1. We found that depletion of FBXW8 by siRNAs consistently increased the basal expression level of endogenous MRFAP1 (Figure 3A). Furthermore, overexpression of FBXW8 caused the decrease of endogenous MRFAP1 in a dose-dependent manner, which was rescued by proteasome inhibitor MG132 (Figure 3B), indicating that FBXW8 regulated the expression of MRFAP1 at posttranscriptional level. Indeed, overexpression of FBXW8 significantly promotes the ubiquitination of MRFAP1, suggesting FBXW8 targets MRFAP1 for proteasome-mediated degradation (Figure 3C). Moreover, 293T cells were treated with cycloheximide (CHX) to block protein synthesis. We found that overexpression of FBXW8 decreased the half-life of MRFAP1 (Figure 4A–4B), whereas knockdown of FBXW8 prolonged it (Figure 4C–4D). In summary, these data demonstrate that FBXW8 regulates MRFAP1 ubiquitination and degradation.

### Cell cycle-dependent degradation of MRFAP1

**Figure 5:**
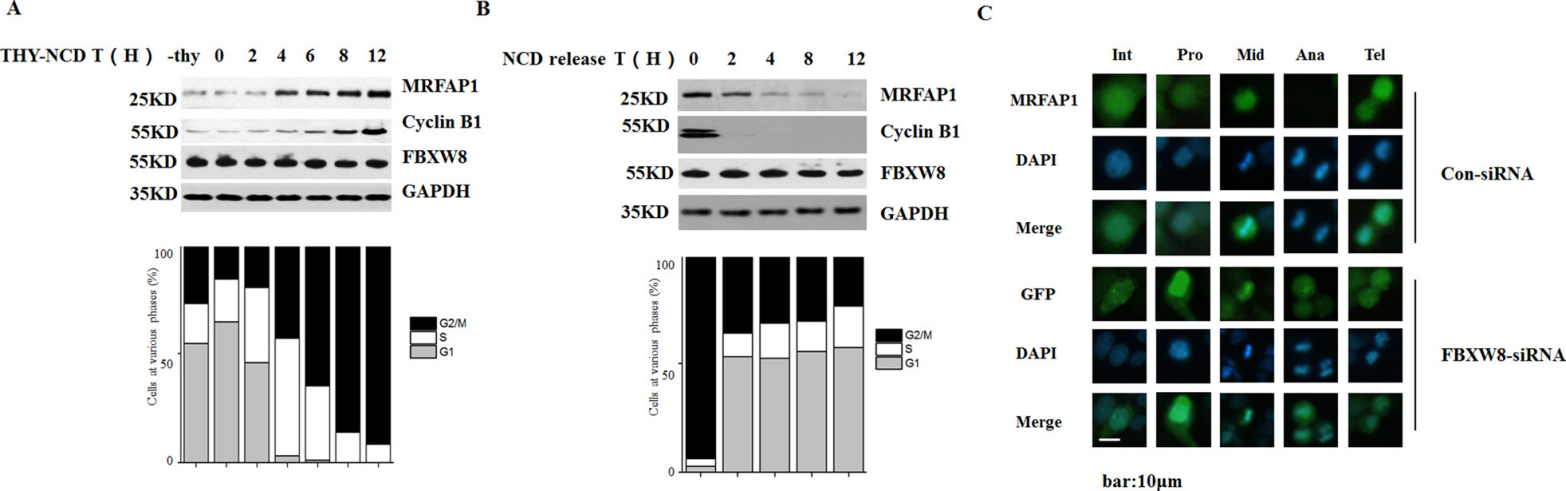
Cell cycle-dependent degradation of MRFAP1 (**A**) HeLa cells stably expressing Flag-MRFAP1 was synchronized by thymidine for 24 hours. Nocodazole was added to the culture after the release from thymidine arrest. The cells were collected at the indicated time and analyzed by FACS. Lysates of these cells were tested by Western blot with the indicated antibodies. (**B**) HeLa cells stably expressing Flag-MRFAP1 was synchronized by thymidine for 12 hours, release for 3 hours, and then blocked by nocodazole for 12 hours. After release from the nocodazole block, the cells were collected at the indicated time and analyzed by FACS. Lysates of these cells were tested by immunoblotting with the indicated antibodies. (**C**) HeLa cells stably expressing Flag-MRFAP1 were transfected with siRNAs targeting FBXW8 or negative control for 36 hours, fixed with PFA and stained with anti-Flag antibody. Various stage of mitosis cells were shown Int (interphase), Pro (prophase), Mid (metaphase), Ana (anaphase), Tel (telophase). Nucleus was stained with DAPI. Bar indicated 10 μm.

Because SCF E3 ligase mediates the ubiquitination of several proteins in specific phases of the cell cycle, we also analyzed the expression of MRFAP1 during the cell cycle. Firstly, we created a HeLa cell line stably expressing Flag-MRFAP1. These cells were synchronized by double thymidine arrest, released, and collected at various time points after release. In addition, nocodazole was added to the culture after the release from double thymidine arrest to activate the spindle checkpoint and prevent exit from mitosis. DNA contents of those cells were monitored by flow cytometry analysis (FACS), and lysates of these cells were tested by immunoblotting. As shown in Figure 5A, the protein level of MRFAP1 significantly increased after cells entering into mitosis. However, the protein level of FBXW8 remained unaltered. Interestingly, the increase of MRFAP1 protein level was even early than CyclinB1, which is known to be accumulated in early mitosis. In order to check how MRFAP1 was regulated when cells released from M phase, HeLa cells stably expressing Flag-MRFAP1, which were synchronized by nocodazole block-and-release, were analyzed by FACS and lysates of these cells were tested by immunoblotting (Figure 5B). As expected, MRFAP1 was highly accumulated in mitosis. However, as cells exited from M phase, MRFAP1 decreased gradually (Figure 5B). In line with these observations, by using immunofluorescence, we found that MRFAP1 was accumulated in metaphase, but completely disappeared in anaphase and reappeared in telophase (Figure 5C, top panel). However, silencing the expression of FBXW8 prevented the disappearance of MRFAP1 in anaphase (Figure 5C, bottom panel). Taken together, the data validate that MRFAP1 is a novel cell cycle-regulated protein and cell cycle-dependent degradation of MRFAP1was mediated by FBXW8.

### Overexpression of MRFAP1 causes mitotic aberrations and cell death

**Figure 6:**
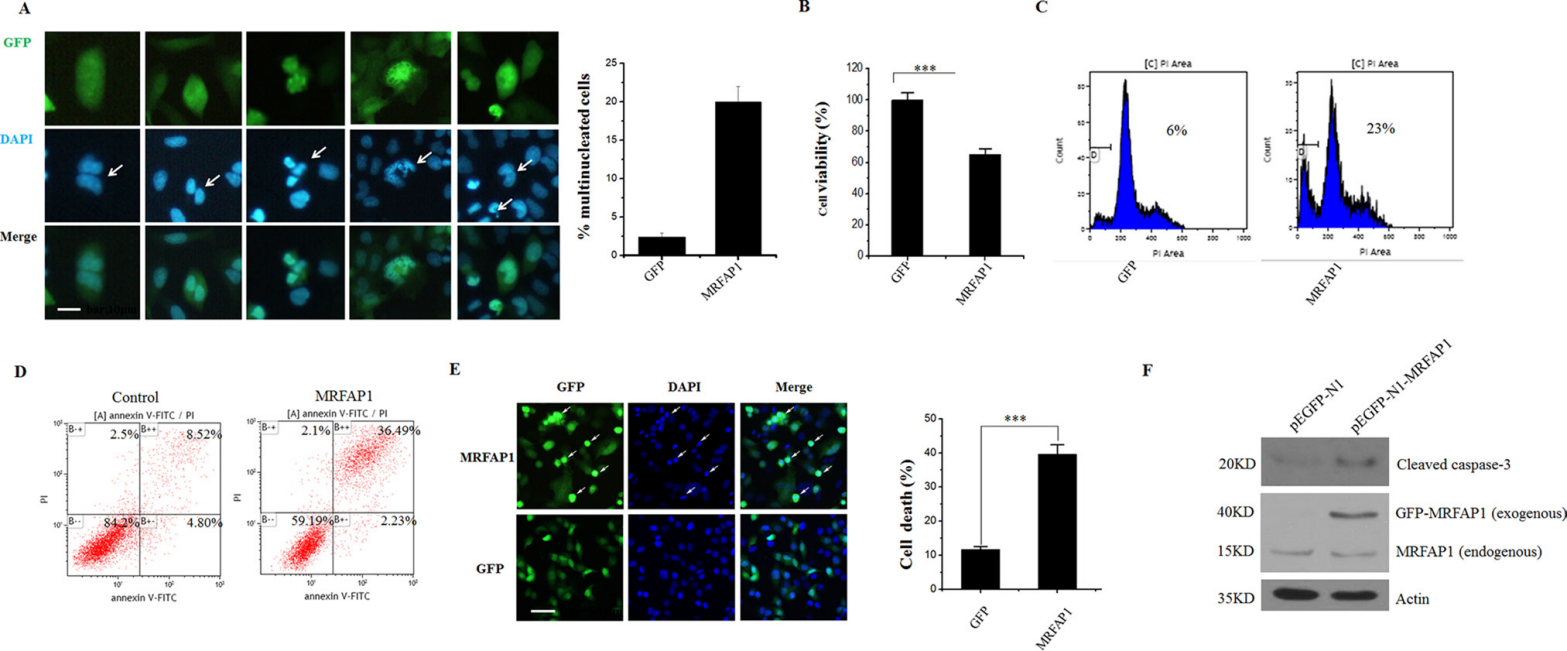
Overexpression of MRFAP1 causes mitotic aberrations and cell death (**A**) HeLa cells were transfected with pEGFP-N1-MRFAP1 plasmid for 24 hours and fixed with PFA. Nucleus was stained with DAPI. Arrows indicated the binuclear cells or multi-lobed nuclei cells. Bar indicated 10 μm. The percentage of the abnormal mitotic cells was calculated. (*n* = 3 for each group). (**B**) HeLa cells were transfected with empty vector or pEGFP-N1-MRFAP1 plasmids for 24 hours, cell proliferation were tested by CCK8 assay. (*n* = 5 for each group). (**C**) HeLa cells were transfected with empty vector or pEGFP-N1-MRFAP1 plasmids for 24 hours, subG1 ratio was analyzed by FACS. (**D**) HeLa cells were transfected with empty vector or pEGFP-N1-MRFAP1 plasmids for 24 hours, and Annexin V positive cells was determined on flow cytometry. (**E**) HeLa cells were transfected with empty vector or pEGFP-N1-MRFAP1 plasmids for 24 hours and fixed with PFA. Nucleus was stained with DAPI Arrows indicated the apoptosis cells. Bar indicated 50μm. The percentage of the apoptosis cells was calculated. (*n* = 3 for each group). (**F**) HeLa cells were transfected with empty vector or pEGFP-N1-MRFAP1 plasmids for 24 hours, cells were then lysed and subjected to western blot with cleaved caspase-3 antibody.

Cell cycle is a precisely regulated process and cell cycle regulated-proteins usually play crucial roles in the regulation processes. Thus, the cell cycle-dependent degradation of MRFAP1 by FBXW8 during mitosis intrigues us to further investigate its biological function in cell cycle control. Aberrant expression of cell cycle regulated-proteins could lead to genome instability, we therefore tested whether MRFAP1 was involved in the regulation of genome stability. As depicted in Figure 6A, overexpression of MRFAP1 in HeLa cells induced mitotic aberrations including binuclear cells, multi-lobed nuclei cells and cell death. In contrast, all these defects were hardly observed in GFP overexpressed HeLa cells. In agreement, HeLa cells with MRFAP1 overexpression exhibited a growth retardant phenotype (Figure 6B). Indeed, HeLa cells with prolonged MRFAP1 expression showed severe cell death by the morphological confirmation, FACS assay determination as well as the activation and cleavage of caspase-3 (Figure 6C–6F), indicating that failure to degrade MRFAP1 in cells produced chromosome instability and eventually lead to cell death.

## DISCUSSION

The identification of substrates for ubiquitin E3 ligases by proteomic assay is extremely difficult, as most substrates are rapidly ubiquitylated and degraded as a result of their association with E3 ligase. The administration of proteasome inhibitors such as MG132 prevented the degradation of substrates might help to improve the identification of substrates of E3 ligases, but without sufficient specificity. MLN4924, a potent and selective inhibitor of NEDD8-activating enzyme, specifically disrupts CRLs-mediated protein turnover [12]. Previous studies showed that substrates of CRLs were highly accumulated upon MLN4924 administration, giving the possibility to identify the substrates of CRLs by Mass Spectrum (MS) [13–15]. In this study, we performed an immunoprecipitation-based proteomics screen with MLN4924 treatment to identify the candidate substrates of Cul7/FBXW8 and demonstrated MRFAP1 as a *bona fide* substrate of FBXW8.

After MRFAP1 has been shown to be one of the most up-regulated proteins after NEDD8 inhibition in multiple human cell lines [10], we tested whether MRFAP1 was regulated by FBXW8. The association between MRFAP1 and FBXW8 was confirmed by means of immunoprecipitation-Western blot as well as exogenous indirect immunostaining. Overexpression of FBXW8 increased the polyubiquitination and decreased the stability of MRFAP1, whereas knockdown of FBXW8 prolonged the half-life of MRFAP1, demonstrating that MRFAP1 is the *bone fide* substrate of Cul7/FBXW8. Substrates of F-box proteins usually involved in cell cycle control and FBXW8 also played a role in cell proliferation, we then examined the expression level of MRFAP1 in cell cycle progress. Interestingly, we found MRFAP1 was a novel cell cycle-regulated protein that accumulated in metaphase, but disappeared thoroughly in anaphase and reappeared in telophase. Importantly, this disappearance could be abolished by FBXW8-depletion, suggesting that FBXW8-mediated MRFAP1 degradation might play a role in the progression of normal cell cycle. Indeed, forced expression of MRFAP1 in HeLa cells caused growth retardation and genomic instability, and eventually leading to severe mitotic cell death.

Although F-box proteins tend to recognize specific degron sequences within target substrates, prior modifications of these degrons are usually required to trigger the interaction between F-box proteins and their substrate proteins, such as phosphorylation, glycosylation and acetylation [4, 16]. To this end, we have also purified MRFAP1 protein and determined the potential phosphorylation sites of it by MS. However, we could not identify any phosphorylation sites (data not shown) and MRFAP1 itself has not been reported to be phosphorylated even in public phosphorylation database, suggesting phosphorylation might not be required for the interaction between MRFAP1 and FBXW8. Nevertheless, further studies are required to clarify the interacting details between MRFAP1 and FBXW8.

Although we have confirmed that MRFAP1 overexpression resulted in decreased cell growth and increased mitotic apoptosis, whether MRFAP1 contributes to DNA re-replication, DNA damage signaling, senescence or autophagy (a phenomena could be observed after MLN4924 treatment) remained completely unknown [1**7**]. Thus, our data has set up the stage for the MRFAP1 function, physiological and pathophysiological significances related researches.

In summary, in this manuscript, by using an immunoprecipitation-based proteomics screen, we identified MRFAP1 was a novel cell cycle-regulated protein and Cul7/FBXW8-mediated destruction of MRFAP1 could be a regulatory component monitoring the anaphase-telophase transition and preventing genomic instability.

## MATERIALS AND METHODS

### Cell culture and treatments

293T and HeLa cells were purchased from the American Type Culture Collection (ATCC). All cells were cultured in Dulbecco’s modified Eagle’s medium supplemented with 10% fetal bovine serum in a 5% CO_2_/95% air at 37°C under saturated humidity. Dulbecco’s modified Eagle’s medium, fetal bovine serum and trypsin were obtained from Invitrogen (U.S.A). MLN4924 (HY-10484, NEDD8-Cullin E3 ligase pathway inhibitor) was purchased from Med Chem Express (U.S.A). Puromycin (P9620, selective agent in eukaryotic cell culture systems), Thymidine (T1895, DNA synthesis inhibitor), MG132 (M8699, proteasome inhibitor), nocodazole (M1404, mitotic spindle inhibitor), cycloheximide (C1988, eukaryote protein synthesis inhibitor) and FLAG M2 Affinity Gel (A2220) were obtained from Sigma (U.S.A).

### Constructs and vectors

MRFAP1 (gene accession number: NM_001272053.1) and FBXW8 (gene accession number: NM_012174.1) constructs were generated by gene synthesis and cloned into the pEGFP-N1, pCDNA3.1(-), and pBABE-3XFlag vector. The construction of retrovirus based stable expression cell lines has been described previously [18].

### CCK8 assay

HeLa cells were inoculated in a 96-well plate 24 hours before transfection. CCK8 (cell counting kit C0038, Beyotime Biotechnology, China) solution was added to each well of the plate 24 hours after transfection. The plate was then incubated for 4 hours in an incubator. The absorbance at 450 nm was measured by using a microplate reader (SpectraMax Paradigm, Molecular Devices, USA).

### siRNAs

FBXW8-specific siRNAs were purchased from RiboBio (Guangzhou, China). The siRNA sequences for human FBXW8 were: No1 5′ GGAGCAUGUUCCUGACACA 3′, No2 5′ GAAGCAAGAUCCUGGUGUA 3′, No3 5′ CAGGAAAGAACUAGGAAGA 3′. 293T-MRFAP1 cell line was plated in a 24-well tissue culture plate for 20 to 24 hours before transfection. For transfection of siRNAs, cells were transfected with 100 nM of siRNA using 1 μL Lipofectamine^®^ 2000 for each well. Cells were then harvested 48 hours after transfection.

### Protein purification, in-gel digestion and Nano-LC−ESI−MS/MS analysis

The whole IP/MS analysis procedure has been described previously [19].

### Immunofluorescence microscopy

Cells were cultured on glass coverslips as described above and then fixed with 4% paraformaldehyde in PBS. Fixed cells were washed with PBS and then permeabilized using 1 % Triton X-100 for 10 min followed by washing with PBS. Coverslips were processed for immunolabeling by blocking with 5% BSA in TBST. Cells were incubated with primary antibodies in 5% BSA in TBST on coverslips for 1 hour. The coverslips were washed with PBS for three times. Primary antibodies were detected with Alexa Fluor 488 or Alexa Fluor 594-conjugated secondary antibodies by incubating on coverslips for 30 min in 5% BSA in TBST. Paraformaldehyde, Triton X-100, and BSA were obtained from sinopharm chemical reagent co.,Ltd (China). Alexa Fluor 488 or Alexa Fluor 594-conjugated secondary antibodies were obtained from Invitrogen (U.S.A). Anti-FLAG antibody (1:1000 dilution, F1804) was purchased from Sigma, U.S.A, Polyclonal anti-MRFAP1 (1:500 dilution, 11639-1-AP) was purchased from Ptglab, Wuhan, Hubei, China, Polyclonal anti-FBXW8 (1:500 dilution, sc-167864) was obtained from Santa cruz, U.S.A. Images were captured by fluorescence microscopy (Bx53, Olympus, Japan).

### Western blot

Cells were harvested and lysed with ice-cold lysis buffer (62.5 mM Tris-HCl, pH 6.8, 100 mM DTT, 2% SDS, 10 % glycerol). After centrifugation at 12,000 g for 10 min at 4°C, proteins in the supernatants were quantified and separated by 10 or 12 % SDS-PAGE, either stained by Coomassie Brilliant Blue or transferred to NC membrane (Amersham Bioscience, Buckinghamshire, U.K.). Primary antibodies used as indicated: monoclonal anti-Flag M2 (F1804, Sigma, U.S.A), monoclonal anti-Flag M2-Peroxidase (HRP) antibody (1:5000 dilution, A8592, Sigma, U.S.A), Polyclonal anti-MRFAP1 (1:2000 dilution, 11639-1-AP, Ptglab, Wuhan, Hubei, China), Polyclonal anti-FBXW8 (1:1000 dilution, sc-167864, Santa cruz, U.S.A). Polyclonal anti-Cyclin B1 (1:2000 dilution, 55004-1-Ap, Ptglab, Wuhan, Hubei, China) or polyclonal anti-Cyclin B1(1:1000 dilution, sc-4073, Santa cruz, U.S.A), polyclonal anti-GAPDH (1:5000 dilution, 60004-1-Ig, Ptglab, Wuhan, Hubei, China)

### Cell cycle synchronization

For nocodazole-thymidine cell cycle synchronization, HeLa cells stably expressing Flag-MRFAP1 were inoculated into a 6-well plate, and treated with 2 mM thymidine for 24 hours, then washed with PBS for 3 times. 0.3 mM nocodazole was added to the culture and the cells were collected at the indicated time for FACS or Western blot analysis. For nocodazole release cell cycle synchronization, HeLa cells stably expressing Flag-MRFAP1 were inoculated into the 6-well plate, and treated with 2 mM thymidine for 12 hours, then washed with PBS for 3 times. After 3 hours, cells were treated with 0.3 mM nocodazole for 12 hours, then washed with PBS for 3 times. After release from the nocodazole block, the cells were collected at the indicated time for FACS or Western blot analysis.

### FACS assay

The whole FACS analysis procedure has been described previously [20].

### Statistical analysis

The Statistical Package for the Social Sciences software version 15.0.1 (SSPS 15.0.1.) was used for statistical analysis. Values were shown as mean ± SEM. Statistical differences were determined by a Student *t* test. Statistical significance is displayed as ^*^p < 0.05, ^**^p < 0.01 or ^***^p < 0.001.
